# Low-grade pelvic masses with spindle cell and fibroblastic proliferation: a case report

**DOI:** 10.1186/1752-1947-1-16

**Published:** 2007-05-02

**Authors:** John Micha, Mark Rettenmaier, Douglas Ellison, John Brown, Bram Goldstein

**Affiliations:** 1 Gynecologic Oncology Associates, Hoag Memorial Hospital Cancer Center, Newport Beach, CA 92663, USA; 2 Hoag Memorial Hospital Presbyterian, Department of Pathology, One Hoag Drive Newport Beach, CA 92658, USA

## Abstract

**Background:**

Abdominal-pelvic masses containing spindle cell and fibroblastic proliferation are very rare. Since scant studies have reported on the pathologic characteristics inherent in this disease, appropriate clinical management is undetermined.

**Case presentation:**

We report on an 87 year-old woman who presented with large abdominal pelvic masses, ascites, ureteral obstruction, and an elevated CA-125 serum level. The patient underwent surgical resection of the lesions and has since done very well. Final pathology revealed a low-grade ovarian tumor with spindle cell and fibroblastic proliferation.

**Conclusion:**

To the best of our knowledge, this appears to be the only reported clinical case of a patient with this rare histology.

## Background

Low-grade pelvic masses with spindle cells and fibroblastic proliferation are very rare, poorly recognized, and not well described in the literature [1]. This histologic subtype has been associated with anaplastic thyroid carcinoma, medullary thyroid carcinoma, but also can reportedly manifest itself as a low-grade fibrosarcoma, malignant fibrous histiocytoma, myxofibrosarcoma, and fibrosing-type fibrosarcoma [2,3].

Myxofibrosarcomas and fibromyxoid sarcomas containing spindle cell and fibroblastic proliferation are not well recognized or classified, and primarily are reported in pathology journals [1,3,4]. Consequently, less is known about appropriate clinical diagnosis and management. Since there appears to be several high and low-grade tumor subtypes exhibiting this histology [5], comprehensive follow-up is necessary to resolve the potential for local recurrence, tumor progression, or metastatic involvement [6]. We describe herein the first reported case involving the diagnosis and clinical management of a patient with low-grade abdominal pelvic masses exhibiting spindle cell and fibroblastic proliferation.

## Case presentation

### History

An 87 year-old (gravida 2, para 2) Caucasian woman was referred to our clinic with a large abdominal pelvic mass, ascites, ureteral obstruction, and a serum CA-125 of 745 U/ML in November 2005. Despite her advanced age, the patient insisted on aggressive management.

The patient underwent a laparotomy, abdominal hysterectomy and bilateral salpingo-oophorectomy. She had approximately 150 cc of clear, straw colored ascites. There was a 20 cm intact, largely solid lower abdominal pelvic mass adherent to the mid and distal sigmoid colon mesenteric serosa, which extended down into the cul-de-sac and left pelvic sidewall peritoneal regions. The patient had a preoperative right ureteral obstruction that also appeared to result from extrinsic pressure related to the 20 cm abdominal pelvic mass. Further examination revealed approximately 6 to 7 cm bilateral solid, whitish ovarian tumors involving the serosa of the mid and upper sigmoid colon mesentery. There were no intra- or post-operative complications and estimated blood loss was 500 cc. The patient is currently doing well clinically ten months postoperatively and adjuvant therapy was not indicated.

### Pathology

Frozen section of the 20 cm abdominal pelvic mass was consistent with a benign fibroma. Pathology further reported that the ovary was white and enlarged, with a smooth and glistening surface. Cross section revealed a unilocular cyst containing yellow translucent fluid. The remainder of the tumor was solid except for small areas of yellow cystic degeneration. There was no identified necrosis. The immunohistochemical findings are significant in that the tumor is only positive for actin, which is not specific but is typically seen in fibrous or smooth muscle tumors. The findings of trichrome and reticulin positivity also favor a fibroblastic lesion.

**Figure 1:**
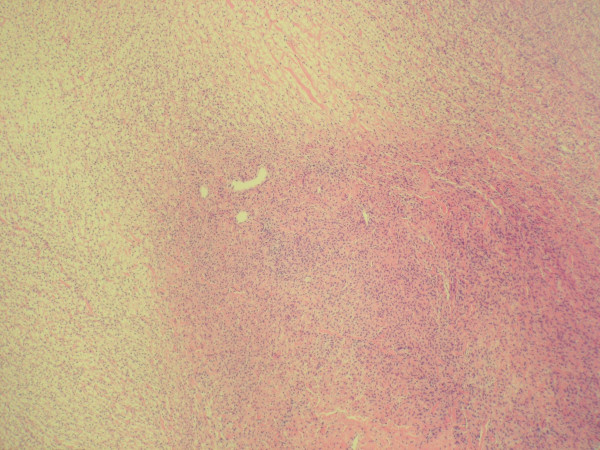
Sections of ovarian mass demonstrate spindle cell proliferation arranged in fascicles with a storiform pattern without cytologic atypia (100×).

**Figure 2:**
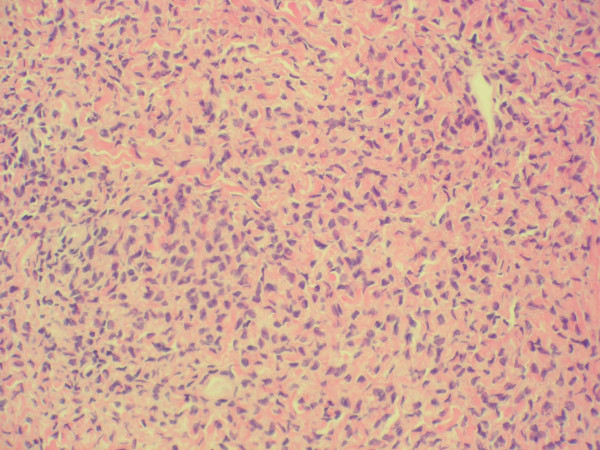
Spindle cells with bland nuclei containing fascicles in a haphazard pattern with variable amounts of collagen (400×).

The case was referred to Stanford Pathology for an independent review. They remarked that the low-grade spindle cell proliferation with fibroblastic features were very rare. The negative immunologic findings exclude more specific markers for smooth muscle, fat tumors, solitary fibrous tumor, hemangiopericytoma, or GIST. They also indicated that these multiple tumors shared the same low-grade appearance and demonstrated a lack of nuclear atypia, increased mitosis, or necrosis. The differential diagnosis included low-grade fibrosarcoma and the solid/low-grade dedifferentiated portion was indicative of an atypical lipomatous tumor (Figures 1 &2).

## Conclusion

We report the rare status of a patient with low-grade abdominal pelvic tumors exhibiting spindle cell and fibroblastic proliferation. This pathologic subtype is very rare and not well described in the literature but may be similar to a low-grade myxofibrosarcoma or fibromyxoid sarcoma [1,3].

Antonescu et al. compared the histologic characteristics of low-grade myxofibrosarcomas with fibromyxoid sarcomas [3]. The myxofibrosarcomas were associated with spindle cells and abundant cytoplasm and the fibromyxoid sarcomas contained more inactive or primitive fibroblastic cells. Myxofibrosarcomas are more often diagnosed in the upper and lower extremities, and associated with greater lesion depth, higher-grade histology, and a propensity for metastases [1].

Fibromyxoid tumors demonstrating a proliferation of spindle fibroblastic cells are uncommonly identified in the pelvic region and can be incorrectly categorized as either sarcomatoid carcinoma or sarcoma [4]. Koh et al. studied two cases of low-grade fibromyxoid sarcoma with MRI, reporting that these sarcomas are rare and often quiescent, yet still have the capacity to metastasize [6]. Hansen et al. described a group of 85 low-grade fibrosarcomas, which contained fibromyxoid, spindle cell or sclerosing epithelioid characteristics [7]. Since this was a pathology study, there was no discussion regarding clinical management for the seven cases that developed recurrent disease.

The woman in our case study initially presented with multiple abdominal pelvic masses and ascites and underwent surgical resection of her lesions. Although final pathology revealed a low-grade ovarian tumor with spindle cell and fibroblastic proliferation, the multiple masses were suggestive of a neoplasm that could recur or metastasize. We recognize that the tumor histology could be classified as a sarcomatous variant. However, since these lesions are often poorly categorized [1] and two pathology institutions were unable to provide a definitive diagnosis, we do not exclude the possibility that this tumor is histopathologically unique.

The proper clinical management for low-grade abdominal pelvic tumors with spindle cell and fibroblastic proliferation should involve surgery and extended follow-up, even if the histology appears benign [5]. We contend that this is critical, particularly since recurrent disease or metastasis may be associated with the histologic diagnosis [1,6,7]. Additional study of the pathologic and biologic characteristics of these tumors should facilitate accurate diagnosis and improve clinical management.

## Competing interests

The author(s) declare that they have no competing interests.

## Authors' contributions

JM originated the study and contributed to the design and drafting of the manuscript. BG compiled the data and significantly participated in the analysis and draft of the manuscript. DE conducted pathologic analysis and review of patient diagnosis. MR and JB significantly contributed to compiling of patient data, analysis, and written manuscript. All authors read and approved the final manuscript.
